# Assessment of PM_2.5_ Exposure during Cycle Trips in The Netherlands Using Low-Cost Sensors

**DOI:** 10.3390/ijerph18116007

**Published:** 2021-06-03

**Authors:** Joost Wesseling, Wouter Hendricx, Henri de Ruiter, Sjoerd van Ratingen, Derko Drukker, Maaike Huitema, Claar Schouwenaar, Geert Janssen, Stephen van Aken, Jan Willem Smeenk, Arjen Hof, Erik Tielemans

**Affiliations:** 1 National Institute for Public Health and the Environment (RIVM), P.O. Box 1, 3720 BA Bilthoven, The Netherlands; wouter.hendricx@rivm.nl (W.H.); henri.de.ruiter@rivm.nl (H.d.R.); sjoerd.van.ratingen@rivm.nl (S.v.R.); derko.drukker@rivm.nl (D.D.); maaike.huitema@rivm.nl (M.H.); erik.tielemans@rivm.nl (E.T.); 2 Province of Utrecht, P.O. Box 80300, 3508 TH Utrecht, The Netherlands; claar.schouwenaar@provincie-utrecht.nl (C.S.); geert.janssen@provincie-utrecht.nl (G.J.); stephen.van.aken@provincie-utrecht.nl (S.v.A.); 3 SODAQ, Bussumerstraat 34, 1211 BL Hilversum, The Netherlands; janwillem.smeenk@sodaq.com; 4 Civity B.V., Handelsweg 6, 3707 NH Zeist, The Netherlands; arjen@wecity.nl

**Keywords:** low-cost sensors, air quality, mobile sensors, PM_2.5_ exposure

## Abstract

Air pollution, especially fine particulate matter (PM_2.5_), is a major environmental risk factor for human health in Europe. Monitoring of air quality takes place using expensive reference stations. Low-cost sensors are a promising addition to this official monitoring network as they add spatial and temporal resolution at low cost. Moreover, low-cost sensors might allow for better characterization of personal exposure to PM_2.5_. In this study, we use 500 dust (PM_2.5_) sensors mounted on bicycles to estimate typical PM_2.5_ levels to which cyclists are exposed in the province of Utrecht, the Netherlands, in the year 2020. We use co-located sensors at reference stations to calibrate and validate the mobile sensor data. We estimate that the average exposure to traffic related PM_2_._5,_ on top of background concentrations, is approximately 2 μg/m^3^. Our results suggest that cyclists close to major roads have a small, but consistently higher exposure to PM_2.5_ compared to routes with less traffic. The results allow for a detailed spatial representation of PM_2.5_ concentrations and show that choosing a different cycle route might lead to a lower exposure to PM_2.5_. Finally, we conclude that the use of mobile, low-cost sensors is a promising method to estimate exposure to air pollution.

## 1. Introduction

Despite a decrease in emissions and ambient levels of pollutants over the years, air quality remains poor in many areas in Europe. As a result, air pollution still forms the biggest environmental risk to human health in Europe, especially in urban areas [1]. One of the main pollutants causing health effects is particulate matter (PM). The health effects of PM are partly dependent on the size of the particles [2]. Particle pollution is grouped into two main categories: parts smaller than 10 μm (PM_10_) and smaller than 2.5 μm (PM_2.5_). The ability of the human body to prevent penetration of particulate matter into human tissue decreases with smaller particle size, causing far reaching health consequences [3]. It is estimated that long-term exposure to PM_2.5_ caused 417,000 premature deaths throughout Europe in 2018. For the Netherlands, the yearly number of premature deaths is estimated at 9900 [1].

Hence, the European Union (EU) has set up limits regarding maximum levels of particulate matter [4]. Annual average PM_2.5_ concentrations should be maintained below 25 μg/m^3^ and PM_10_ concentrations should remain below 40 μg/m^3^. However, even below these limits, adverse health effects might occur [3], and according to the WHO “no threshold has been identified below which no damage to health is observed” [5].

EU legislation requires member states to monitor PM levels with official measurement methods, as defined in the European Air Quality Directive (2008/50/EC) [4]. In the Netherlands, monitoring takes place using the National Air Quality Monitoring Network (Dutch: Landelijk Meetnet Luchtkwaliteit (LML)) [6]. In addition, air quality assessment is performed using an extensive set of modeling within the framework of the National Air Quality Cooperation Program (NSL) [7,8,9].

The costs of a national system with high-quality reference stations are high. Consequently, the network is sparse, limiting the ability to capture spatial variability in air pollutant concentrations, which cannot always be compensated for by additional modeling work.

To improve the spatial and temporal resolution of the existing air quality monitoring network, alternative air quality monitoring solutions, such as the deployment of a large number of low-cost sensors, are suggested [10]. Low-cost sensors might be added to the existing network to improve its spatial representation [11,12], and do not need to be operated by official institutions [13,14], which makes it an attractive option for governments and citizens alike. Interest in the use of low-cost sensors is rapidly increasing, for a large part driven by their low cost and ease of operation. In Europe, many thousands of low-cost PM sensors are already deployed, primarily through initiatives like “Sensor.Community”. In the Netherlands, several experiments with low-cost sensors have also been performed [15]. Associated sensor data are available at sensors.rivm.nl. Wesseling et al. (2019) analyzed these stationary sensor measurements and concluded that the rise of citizen science and increasingly better performing low-cost sensors will change the role of Environmental Protection Agencies (EPAs) significantly in the coming years [13]. In recent years, several studies have given an extensive overview of the use of low-cost sensors, their calibration options and other technical considerations [16,17,18]

Another reason for the increasing interest in low-cost sensors is their potential for personal exposure monitoring [17,19]. Typically, estimates for exposure are based on modeled PM concentrations at home addresses or zip codes. However, these studies cannot account for exposure to pollutants in other locations, or, for instance, while commuting. Low-cost sensors offer an attractive method to assess individual exposure to particulate matter as they can be used as portable sensors [20]. However, typical studies on personal exposure characterization using portable sensors have few participants, which makes it difficult to use these data to improve the spatial resolution of PM measurements. When there are many individual participants in an experiment new challenges arise as the quality of the measurements may vary significantly [14].

In this study, we aim to improve the spatial and temporal resolution of PM_2.5_ measurements by deploying a large number of low-cost PM sensors mounted on bicycles. Moreover, we specifically investigate whether it is possible to estimate exposure to traffic related PM_2.5_ using these low-cost sensors. This is important because it has been suggested that traffic-related emissions, particularly soot emissions, are most harmful to human health [21].

In the Netherlands, more than one-quarter of all trips are made by bicycle [22] while the average number of bikes per person in 2020 was 1.3 [23]. Cycle paths in the Netherlands are often part of a dense network, particularly in urban areas. Because of this dense network, there is a large variety in the type of cycle paths. Some cycle paths are adjacent to major roads for motorized traffic, while others are completely segregated, e.g., through the woods. This makes it possible to compare different cycle routes with different expected contributions of traffic-related PM. Modeling and empirical research show that PM concentrations in the Netherlands are highest around major transportation routes [24,25,26,27]

Our study has three main objectives. First, we will discuss the quality and validity of PM_2.5_ measurements using low-cost sensors on bikes. Secondly, we will use these measurements to estimate exposure to traffic related PM_2.5_ during typical commutes. Finally, we will reflect on the use of mobile, low-cost air quality sensors and their potential to estimate personal exposure to PM.

## 2. Materials and Methods

For this study, we use PM_2.5_ sensor data obtained from the “Sniffer bike” project for the year 2020 (in Dutch: https://snuffelfiets.nl/ (accessed on 25 May 2021)). In this project, around 500 cyclists used the Sniffer bike sensor kit to measure PM_2.5_ concentrations. The Sniffer bike is a co-operation between citizens, provinces/municipalities, private companies, and a research institute. The province of Utrecht provides the financing, the project- and program management, and platform for the community. Within the organization of the province, multiple policy areas are involved (e.g., health, mobility and data, knowledge, and innovation). Local volunteers are being involved by municipalities, platforms, such as cyclist associations, groups of cycling friends, and through their jobs as bicycle couriers. SODAQ, an Internet of Things company, participates as a partner for the delivery and maintenance of the sensors. The company Civity is responsible for the data management and the data platform. RIVM, the Dutch National Institute of Public Health and the Environment, validates and analyses the collected data.

**Figure 1:**
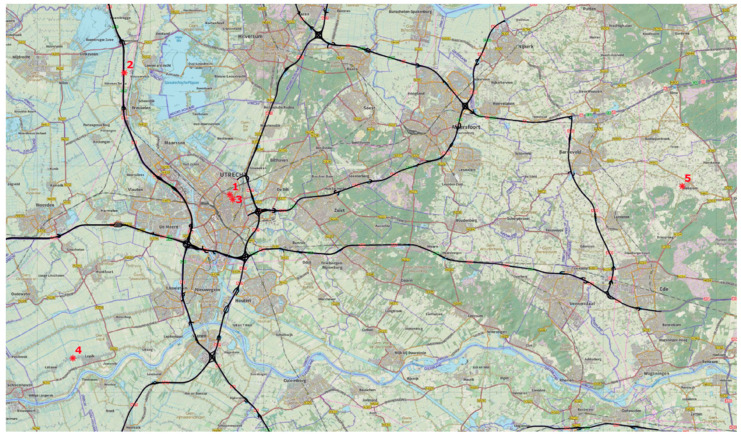
Area of the province of Utrecht. The “*” markers indicate the locations of official air quality measurements (PM_2.5_) at Kardinaal de Jongweg (1), Breukelen (2), Griftpark (3), Cabauw (4) and Wekerom (5). Source map: pdok.nl (accessed on 25 May 2021).

The area of the Sniffer bike project in Utrecht is shown in Figure 1. The province of Utrecht has a very high population density and consists of several urban centers, most notably the city of Utrecht (population of ~360,000; located in the west) and Amersfoort (population of ~158,000; located in the northeast). The center of the province is dominated by National Park “De Utrechtse Heuvelrug”, the second largest forest area in the Netherlands. There are many relatively quiet trails (non-motorized) for walking and biking in this national park. As a result, because of the urban centers and the presence of a national park, there is a large variety of different cycle paths in the study area. Sniffer bike sensors are not geographically restricted to the province of Utrecht; they have been deployed elsewhere, and people are allowed to use them wherever they are. However, for this study, we concentrated on the measurements in the province of Utrecht as the Sniffer-bike project originated there, with most measurements performed there. Of the approximately 25.5 million Sniffer bike measurements in the Netherlands in 2020, about 20 million were located in the area of the province of Utrecht.

The sensor kit in the Sniffer bike contains several sensors and additional hardware, including:

- Sensirion SPS30 dust sensor for air quality (PM_1_, PM_2.5_, and PM_10_);
- Bosch BME680 temperature and humidity sensor;
- Accelerometer to collect data about the pavement quality (LSM303AGR—part of SODAQ Sara SFF board);
- GPS and time observations to display the cycled route (u-blox EVA-M8M—part of SODAQ Sara SFF board).

**Figure 2:**
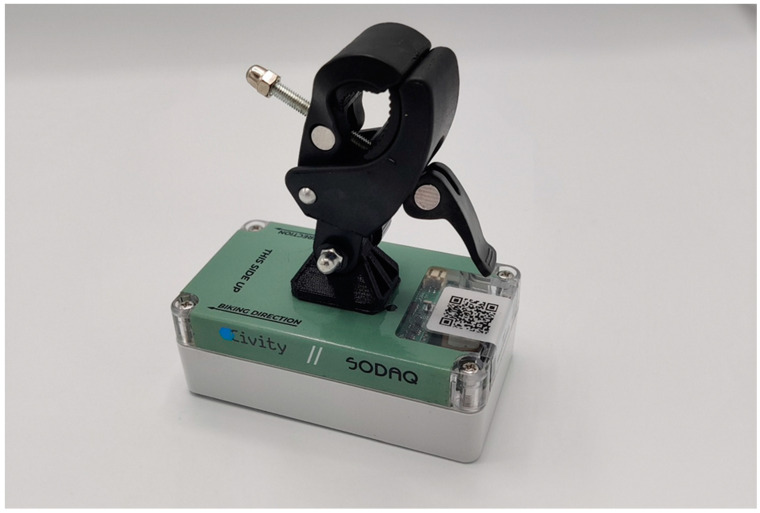
Housing of a Sniffer bike sensor kit.

The housing is mounted onto the handlebar of the bicycle (see Figure 2). The housing contains the sensors, the battery and the communication unit. The communication is facilitated via Long Term Evolution (4G), category M1 (LTE-M), which is a wireless communication standard which belongs to the category of low-power wide-area networks (LPWAN), enabling to connect devices that need small amounts of data, low bandwidth, and long battery life. The IMEI-number is the unique identification of the sensor kit, used throughout the whole process. Being on a mobile platform, the power of the sensors is provided by batteries that have to be charged before the trips. In theory, a charged set of batteries should allow for up to 8 h of measurements, more than enough for daily use. In practice, this was an upper limit and not all users kept the batteries sufficiently charged. A guide on how to build your own “Sniffer bike” sensor kit (of the initial pilot tests) is shown on the website of the manufacturer [28].

**Figure 3:**
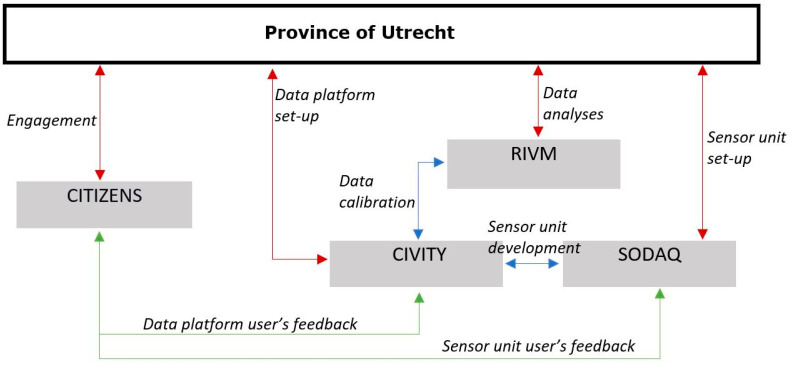
Data flow in the Sniffer bike project.

The sensor kit is only active when moving. Every ten seconds a sample of air is taken, and the measurements, including location, are sent to a server at the company Civity. The raw data are then sent to RIVM where it is stored into a dedicated InfluxDB data base and subsequently calibrated. The calibrated data are finally POSTed back to Civity (see Figure 3 for an overview of the data streams). During the project, we found that the GPS had issues getting a fix on the position when the bikes passed through the woods. In these cases, concentration data were provided without a position and without the possibility to estimate the speed. Therefore, these data could not be used in the analysis. The data of the temperature sensors will be analyzed in a separate project. The other data have not been used so far. The price of the measuring kit is roughly 500 Euros. On top of this, there are costs for creating a data infrastructure and a data portal for participants. The present project was sponsored by the province of Utrecht.

For the calibration of the sensor data, we use official measurements. RIVM performs official measurements of the PM_2.5_ concentration in accordance with the EU directive 2008/EC/50 [4]. This is done using four stations from the LML network. The hourly PM_2.5_ measurements are performed with the Met One BAM 1020 [29], for which was shown that the measurement results are equivalent to those of the reference method. The hourly results of the measurements, additional information, and historic data are available at www.luchtmeetnet.nl (accessed on 25 May 2021). Figure 1 shows the locations of the official measurements used in this study.

The dust sensor used in this project is the Sensirion SPS30 [30]. Based on our previous experiences with this sensor, and other studies [31], we know that this sensor is not very good at measuring the coarse fraction of PM (PM_10_), but reasonably able to estimate PM_2.5_-concentrations. Therefore, in this study, we only consider the concentration of PM_2.5_.

### 2.1. Clean-Up and Calibration of the Sensor Data

In this section, we describe the clean-up and calibration of the data. First, we describe the filtering of the data that took place. Next, we discuss how the sensors were calibrated using information obtained by co-locating a set of sensors on official measuring locations. Finally, we discuss the variability and uncertainty of measurements obtained while cycling (i.e., the effect of having to deal with mobile sensors).

The sensor data were first processed to filter out potential improper use of the sensor (mainly indoor use) or malfunctioning sensors. This was necessary as it was not possible to monitor the correct functioning of each individual sensor during the campaign. In the processing, all speeds of the sensor kits were estimated to filter out unreasonably high speeds or stationary use. All measurements obtained at speeds higher than 45 km/h and lower than 5 km/h were excluded. The upper limit corresponds to the maximum speed possible using a “Speed Pedelec” e-bike. After applying this filter, almost all measurements located on highways and train tracks disappeared. To exclude stationary use, we applied a lower speed limit of 5 km/h. This also got rid of most indoor measurements, as these are practically stationary. After applying these speed limits, roughly 60% of the measurements were accepted as valid for further processing.

**Figure 4:**
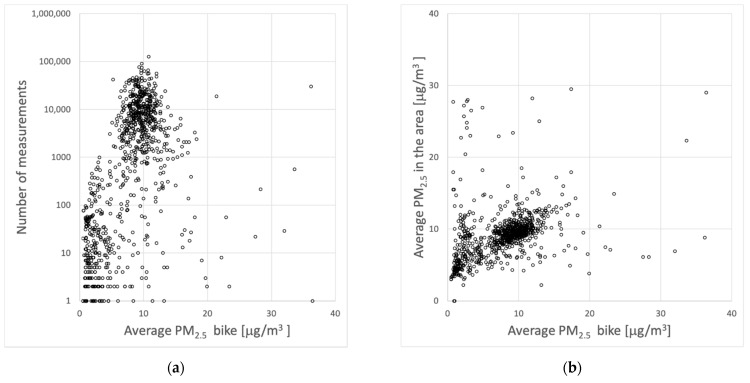
(**a**) The number of valid measurements of sensor kits as a function of the average concentration measured by that kit. (**b**) Average concentrations of the official measurements during the hours when each specific kit was active and compared to the average of that kit.

A small number of sensors provided large numbers of measurements. Figure 4a shows the relation between the number of measurements and the average calibrated concentrations reported by the sensors. Figure 4b shows the relation between the average official concentrations in the Utrecht area and the average calibrated concentrations reported by the sensors. So, for each sensor kit, we took the average concentrations of the official measurements during the hours when that specific kit was active and compared this to the average of the kit itself.

Some sensors report values that are relatively low or high with respect to the bulk of the measurements (which scatters around 10 μg/m^3^). The very low and high values may be correct but could also indicate issues with the sensors. For instance, there is a group of sensors showing very low average concentrations, below 3 μg/m^3^. These results hardly correspond to the average concentration in the region at the same hours of these sensor measurements. The average concentration in the area was 2 to 8 times higher than the sensors, suggesting malfunctioning sensor kits or improper use. For example, very low values are reported if the inlet of the PM sensor is covered. On the other hand, one sensor reported about 30,000 measurements with an average concentration of 36 μg/m^3^. As the average concentration in the area during the hours measured by this sensor was only 9 μg/m^3^, this suggested that the sensor was not functioning properly. The suspect data (average concentrations below 3 μg/m^3^ and the one sensor with an average of 36 μg/m^3^), is observed in 21% of the deployed sensor kits reporting valid data, but represents only 0.7% of the total data that were available after initial filtering. Excluding these data from the analyses did not seriously impact any of the results and conclusions.

**Figure 5:**
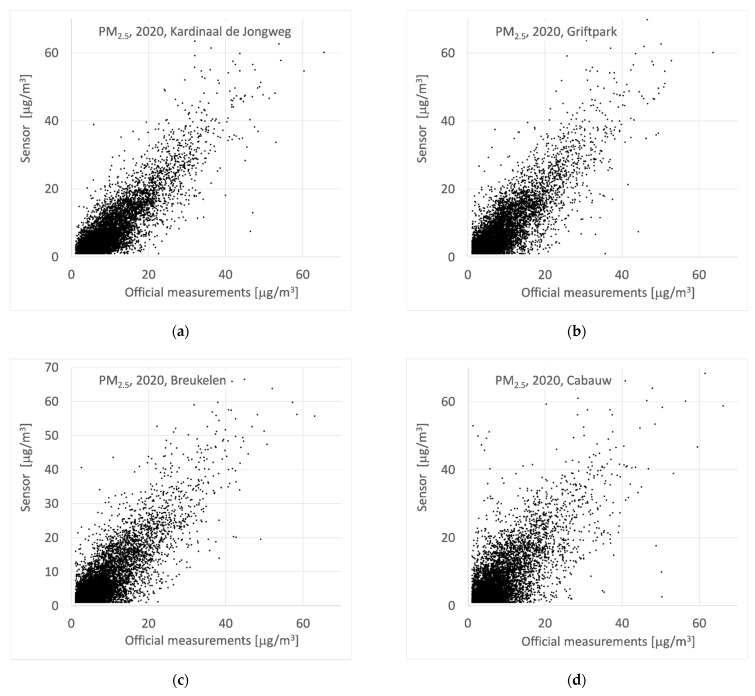
(**a**) Results of official measurements and nearby/co-located sensors at Kardinaal de Jongweg; (**b**) results of official measurements and nearby sensor at Griftpark; (**c**) results of official measurements and nearby/co-located sensors at Breukelen; (**d**) results of official measurements and nearby/co-located sensors at Cabauw.

After the cleanup and filtering, the sensor data were calibrated. Calibration is needed because concentrations reported by low-cost sensors are affected by several factors, such as meteorological circumstances (e.g., humidity). For this calibration, we relied on data from co-located sensors at three official reference stations in the province of Utrecht. A fourth official location was close enough to co-located sensors to also participate in de measurements. As a first check, we compared the official hourly PM_2.5_ measurements with the average values of all sensors passing by that location (within 1 km) and with sensors co-located at that station (Figure 5).

At a first glance, the comparison between official measurements and the results of the sensors is quite satisfying. However, when looking in more detail, there are non-trivial differences between sensors and official measurements, especially at concentrations below 15 μg/m^3^. There are correlations between the absolute concentrations, the humidity and the ratio between sensor and official measurement. We did not fit correction factors as a function of environmental parameters but decided to estimate a general hourly correction factor based on the ratios observed at the locations with co-located sensors. As we are looking at measurements in a relatively small and uniform region in the Netherlands, we expect such a general correction factor to be applicable in the whole region. We write the hourly calibration as $C_{sensor,\;cal}=\beta \;C_{sensor}$, with the hourly correction factor defined by:

(1) $$\beta =\frac{1}{n}\;\sum \frac{C_{LML}}{\bar{C_{sensors}}}$$

here $\bar{C_{sensors}}$ is the combination of values of sensors co-located at official locations and sensors reporting data while passing within one km of official locations. If *n* < 2, then only one station is too limited to estimate a calibration for the whole area and we take β=1. To assess the uniformity of the concentration field in the area, we calculate the standard deviation $\sigma_{LML}$ of the available official measurements. A large standard deviation indicates a limited representativity of the correction value for the whole area. We impose that 0.5 ≤ β≤5.0 and for $\bar{C_{sensors}\;}>15$ we only apply the hourly correction if it has a relatively small uncertainty: $\sigma_{LML}<0.15\;\bar{C_{LML}}$. In practice, this means that the raw data are used if the calibrations at the available official locations differ too much. We explicitly do not only use the single calibration factor closest to a sensor measurement as this creates a dependency on one co-located sensor and one official measurement. Less representative measurements by either one will then directly impact all sensors in that area. Having multiple sensor kits at all the locations of the official measurements resolves this. A drawback of using a common hourly correction factor for all sensors is that specifics of the sensors, such as aging or malfunctioning, are not considered. However, users of the sensor kits had an app that provided them with information about their trips and measurements. In case they observed suspicious measurements or malfunctions of the sensor, they reported this and were supplied with a new or repaired sensor (if possible). However, given the amount and geographic distribution of the sensors during the project, there was no realistic alternative to our calibration approach, like repeated individual calibration of the sensors.

Comparing the raw sensor data to the official measurements results in standard deviations of the differences between 4 and 6 μg/m^3^. There is an average bias of −1.2 μg/m^3^ and 95% of the sensor data lies within ±4 μg/m^3^ of the official data (95% CI of 8 μg/m^3^). The distribution of the differences between hourly results of sensors and official measurements shows relatively high and long tails, indicating the potential of sensors to report substantial deviations from the real values. Using the calibration system described above, comparing all available calibrated co-located sensor data to official data results in differences with a 95% CI of 6 μg/m^3^ and a bias of 0.3 μg/m^3^. Of course, this is partly because the stations themselves were used in the calibration. It should be noted that the locations of Kardinaal de Jongweg (along a moderately busy open street) and Griftpark (in an urban park) are just 500 m apart. The yearly average concentrations at these locations differ only 1 μg/m^3^, roughly 10%. Given the close proximity, the data from the sensor co-located at Kardinaal de Jongweg is also combined with the official data at Griftpark. So, this sensor contributes to two calibration factors, one as if it is located at a street location and another as if it is at an urban background location. Of course, other sensors, passing by in the neighborhood of official locations also contribute to the calibration factors.

**Figure 6:**
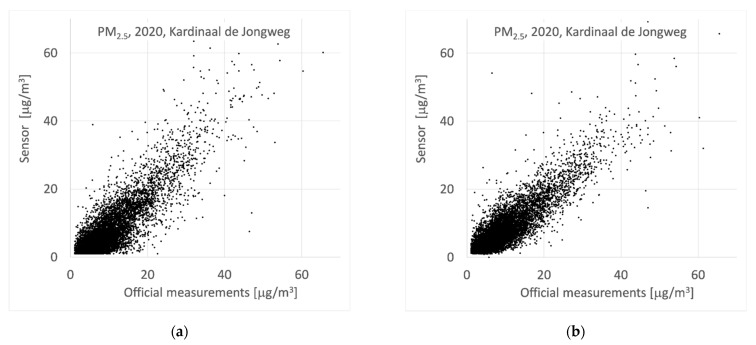
(**a**) Results of official measurements and co-located/nearby raw (uncalibrated) sensors at the location “Kardinaal de Jongweg”; (**b**) results of official measurements and co-located/nearby calibrated sensors at the location “Kardinaal de Jongweg”.

To test the calibration algorithm, we calculated hourly correction factors for the sensors *without* using the official data at location “Kardinaal de Jongweg” and then applied this factor to the sensor located at “Kardinaal de Jongweg” (Leave Station Out, LSO). The results are shown in Figure 6. The underestimation of the concentration at lower concentration levels and higher relative humidity’s was substantially reduced. Overall, the correlation looks better, although it only slightly improves from 0.86 to 0.87.

The test was performed for all co-located sensors. For each location, the hourly correction factors in the year 2020 were calculated excluding the location itself. These correction factors were subsequently applied to the sensor at that location and the results were compared to the official measurements. When each sensor is corrected using data from the other co-located sensors in the area (Leave Station Out calibration) the differences between sensor data and official measurements decrease and the bias is corrected for. The short distance between Kardinaal de Jongweg and Griftpark contributes to a good performance in the LSO test. Therefore, we have tested the LSO calibration for these stations, using only calibration factors obtained at Cabauw and Breukelen (both well outside of the city). For both the raw sensor data and the LSO calibrated data the mean concentrations, the Mean Average Error (MAE), the Pearson correlation and the Root Mean Square Error (RMSE) have been calculated. The results of the calibration-tests are summarized in Table 1.

As mentioned before, an important benefit of the calibration is the reduction of the bias, which practically disappears. On average, the Pearson correlation decreases from 0.81 to 0.80. The MAE and RMSE improve by the calibration, for all but one case. In the analysis of the bike data, for an individual sensor in the city of Utrecht the calibration will be based on information of official measurements that were excluded for the purpose of testing the calibration. As a result, the calibration is expected to perform better than shown in these tests. Based on the tests described above, we assume the 95% CI of the calibrated hourly sensor measurements to be in the order of 6 μg/m^3^. As the hourly concentrations of the co-located sensors are based on many 10-s measurements it is clear that the observed uncertainty must be of a systematic nature. This suggests that the estimate of the uncertainty will also hold to a large extent for measurements that do not last a full hour but only 30 or 15 min.

The Leave Station Out calibration also allows estimating the uncertainty of the calibration regarding the yearly average concentrations. Throughout the whole year of 2020, the average bias between the yearly average concentrations by the sensors (after calibration with the LSO method) was 0.1 μg/m^3^ and the standard deviation of the differences was 1.1 μg/m^3^. This is interpreted as a 95% CI of 2.2 μg/m^3^.

### 2.2. Scatter of Measurements While on a Bike

To assess the quality of measurements while the sensor kits are mounted on bikes and moving, we look at the values of different sensors when they coincidently pass through the same 50 × 50 m^2^ grid cell in the same hour. The estimate of the speed is based on the last two positions. This implies that data of bikes that have just stopped at an intersection or at traffic lights are also considered. We require at least two sensors to report data. This results in about 5000 h/cell combinations with co-incident sensor measurements in 2020. First, we take the average of the measurements of each sensor in the cell and then calculate the standard deviation of these averages in the cell. The scatter between co-incident measurements is, with roughly 25–30%, comparable with that between LML and sensors. We therefore conclude that the scatter in measurements does not substantially increase when the sensors are used on (moving) bikes. If the duration of a bike trip takes in the order of one hour, we assume an uncertainty in the average concentration as derived above for the co-located sensors, with the 95% CI of the mobile measurements in the order of 6 μg/m^3^. As discussed above, we assume the uncertainty to be roughly similar when there are only measurements during a substantial part of an hour.

### 2.3. Variations of Measurements during Bike Trips

The above uncertainties are all for hourly average concentrations and differences between individual sensors. In order to estimate PM_2.5_ concentrations during bike trips, the variations of individual sensors over smaller time scales than hours are important.

The 10-s measurements during a bike trip are mainly influenced by (1) the changes in the overall PM_2.5_ concentrations in the province of Utrecht; (2) the overall calibration of the sensors; (3) random fluctuations in the sensors themselves; and (4) the local sources surrounding the bikes (mainly traffic). For our analysis, it is important that the effects of (1), (2), and (3) are small compared to those of (4) or, alternatively—that we can prevent these effects from influencing the analysis.

To check the effect of large-scale variations of the concentrations in the whole province on the results of individual bike trips (issue 1), we excluded data in hours where the average variation in PM_2.5_ concentration measured at the official locations in Utrecht city exceeded 2.5 μg/m^3^ per hour. Given that bike trips, on average, take 17 min, this implies that we do not expect a significant influence of changes in the general concentrations in the province on the estimated concentrations along a bike trip. As a result of this constraint, for 6.3% of the bike trips, the concentrations along the trip were not included in the sensitivity analysis (see Section 3.2). Evidently, incidental fast changes in concentration cannot be excluded but these do not occur often. Moreover, during longer bike trips, there may be an effect of changes in the general background concentration.

As discussed in Section 2.1, the overall calibration (issue 2) can be addressed using the co-located sensors. We use the hourly calibration factors determined using all available co-located sensors to correct all sensors in the field. As a result, the differences in concentrations measured along bike trips are also calibrated.

Data of measurements during several bike trips suggested that the short-term random fluctuations of the sensors (issue 3) are quite limited. To verify this, we looked at several months of data of the sensors co-located at official locations as these sensors produced data all the time. As bike trips take on average 17 min, just over 1000 s, the standard deviations of every series of 100 consecutive datapoints were calculated. With an average standard deviation of roughly 1 μg/m^3^, the random fluctuations of the 10-s measurements are indeed quite low.

From the above we conclude that fluctuations in measured concentrations exceeding 0.5–1.0 μg/m^3^ on a short time scale of 10–30 s are most likely caused by the varying ambient concentrations during a bike trip. At this time scale, the concentrations around the bikes may change substantially. Even at moderate speeds of the bike of 10 km/h it travels some 2.8 m per second. The time between two measurements is enough to cross a street with busy traffic. Between three reported measurements by the sensor kit, it probably traveled (at least) almost 100 m. This represents a large enough distance to travel from, for instance, a relatively quiet street in a residential area into a street with substantial amounts of traffic at short distance from the bike.

**Figure 7:**
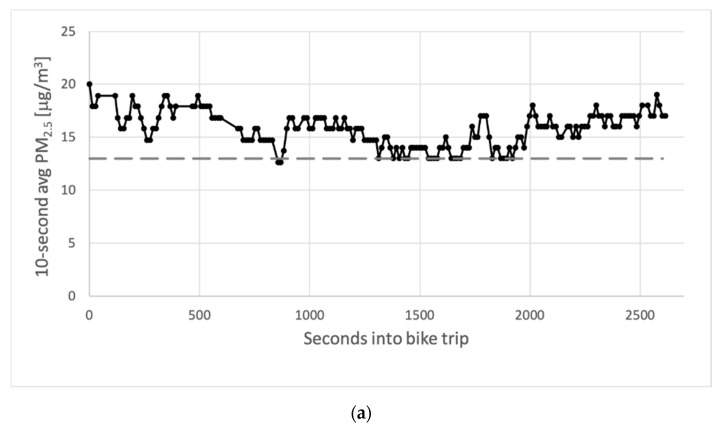

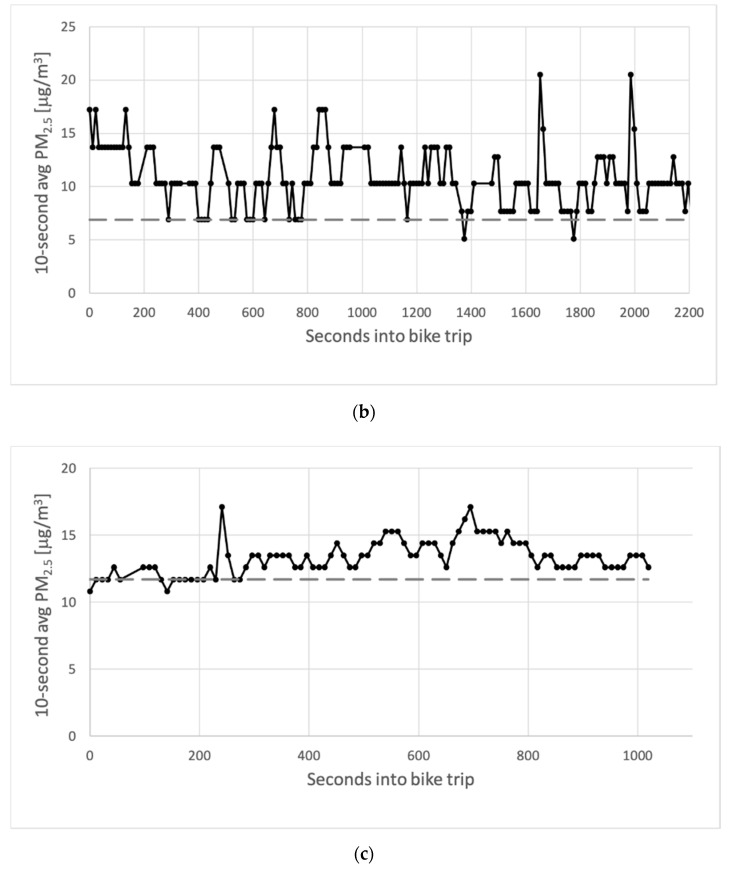
Several typical time series of measurements during bike trips. Concentrations in μg/m^3^ are plotted as a function of the seconds passed since the start of the trip. The dotted lines indicate the level of the assumed background concentration. (**a**) Measured concentrations during a bike trip on 21 January 2020; (**b**) measured concentrations during a bike trip on 23 May 2020; (**c**) measured concentrations during a bike trip on 10 August 2020.

As the measured concentrations are calibrated using the hourly calibration factors, changes in these calibration factors during bike trips also influence the estimated contributions from traffic. Assuming the correction factors to change linearly within hours, the standard deviation of changes in the applied calibrations is in the order of 15%, leading to an estimated (systematic) 95% CI of 30%. The fluctuations of the sensors between subsequent measurements lead to a random uncertainty of 1/sqrt(n), with *n* the number of measurements during a bike trip. Several typical time series of measurements in bikes are shown in Figure 7.

In short, we have calibrated sensors using co-located sensors at official stations. Using a “Leave Station Out” approach, we find that systematic biases in the sensor data are corrected for and measurements are more in line with official measurements. Moreover, by checking data from sensors that are, by chance, simultaneously present in the same grid cell, we show that differences in sensor data for different sensors (moving on bikes) are limited. Finally, we checked the fluctuations in measured concentrations at a higher temporal resolution than for the hourly values and find that there generally no large deviations from the overall trend in concentrations.

### 2.4. Estimation of Exposure to Traffic-Related PM

In order to estimate the exposure of the bikes to emissions of the surrounding traffic we take the concentration profile during a bike trip and assume that at least part of the trip will be at locations where there is little traffic nearby. Typically, a trip on a bike in the Netherlands starts at the home address, most of which are not located directly on a street with busy traffic. Moreover, many office spaces, schools, and other likely destinations (or starting points) of a trip on a bike are not directly on a street with busy traffic. Many bicycle trips will pass through or go along a number of streets and roads with substantial traffic. Most bicycle trips do not last very long and are limited to several kilometers. We take the lowest measured concentration (assuming no uncertainty) during the trip as an estimate for the local background concentration during the bike trip. In practice, there are complications and we cannot simply take the lowest value. At the start of a trip, the sensor needs some time to start measuring properly, especially when the sensor kit is taken from an indoor location, a substantial change in temperature and humidity at the start of the measurements can occur. Depending on the circumstances, we estimate that it may take up to a few minutes for the sensor kit to sufficiently cool down or warm up. Apart from the potential initialization issues, we have to take the uncertainty and scatter of the sensor values into account. Due to these effects, the lowest measured value will probably be lower than the background level we are looking for. We therefore do not use the lowest measurements, but use the 10% percentile of the concentrations as an estimation for the background concentration. Taking either the 5%, 10%, or 15% percentile results in a difference of roughly 0.4 μg/m^3^ in the derived average exposure to traffic emissions between each step. This difference is treated as an indication of the systematic uncertainty of the approach. The possible variations in correction factors during bike trips lead to a systematic uncertainty of 15% in the estimated exposure. On top of this, the fluctuations of the sensors at the smallest time scale add a random uncertainty. However, the observed random variations between subsequent measurements of the sensors of 1 μg/m^3^ will, given enough measurements in a bike trip, not contribute significantly to uncertainties of the estimated exposure.

## 3. Results

### 3.1. Number of Bike Trips and Average Concentration Measured in 2020

**Figure 8:**
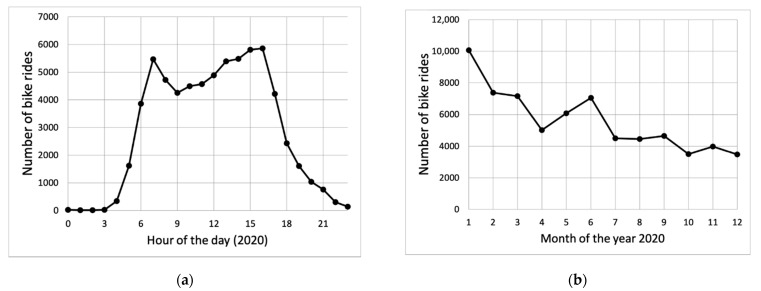
(**a**) The number of bicycle trips with valid data for the present analysis in 2020 as a function of the hour of the day and (**b**) the number of bicycle trips with valid data as a function of the month of 2020.

After the clean-up and filtering of the data in 2020, there remain just over 8.1 million valid data points. There are almost 68,000 bicycle trips with valid data in 2020, and there were valid trips on every day of the year. The number of bicycle trips is not uniformly distributed over the day. There are relatively more trips in the morning and late afternoon and there are practically no trips during the late evening or night, as is shown in Figure 8. There is a steady decrease in the number of trips over the first part of 2020. During the last six months, the number of valid trips was almost constant. The average time spent on a ride was 17 min, which is a reasonably typical length for a trip on a bike in the Netherlands.

**Figure 9:**
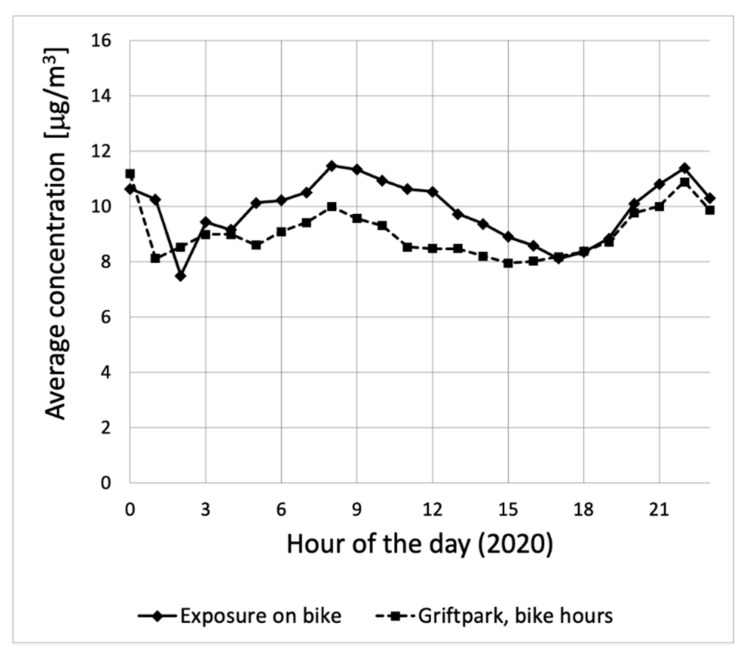
The average PM_2.5_ concentrations during bicycle trips in 2020 as a function of the hour of the day. The average PM_2.5_ concentrations at the urban background location “Griftpark” are also shown for comparison.

The concentrations measured by the bikes are in part due to varying PM_2.5_ concentrations in the whole province. The sensors on the bikes measure these background concentrations and on top of those also the local contributions of the surrounding traffic. The hourly average concentrations measured by all the bikes in 2020 are shown in Figure 9 as a function of the hour of the day. The figure also shows the average concentrations measured at the urban background location “Griftpark” in the city of Utrecht. Here, only hours with valid bike measurements were considered.

The average concentration measured by the sensors during all valid bike trips in 2020 was 9.9 μg/m^3^. During the day, the average concentrations measured by the bikes are up to 2 μg/m^3^ higher than the average urban background concentrations in the city of Utrecht. Later in the afternoon, the average concentrations measured by the bikes and at the urban background location are practically the same. This was not necessarily the case in the whole area being studied, as the location in the city of Utrecht is not representative for the whole province of Utrecht. The scatter in the exposure on bike is large, as bike measurements are performed all over the province, not only in the city of Utrecht. Therefore, the two curves in Figure 9 cannot be compared directly. They also do not provide a general indication of the difference in concentrations measured on the bikes and at Griftpark. As there are only three (urban) background locations with official PM_2.5_ measurements in the area, it is not possible to estimate the background concentrations at every location by comparing the measurements of the bikes to nearby official data.

**Figure 10:**
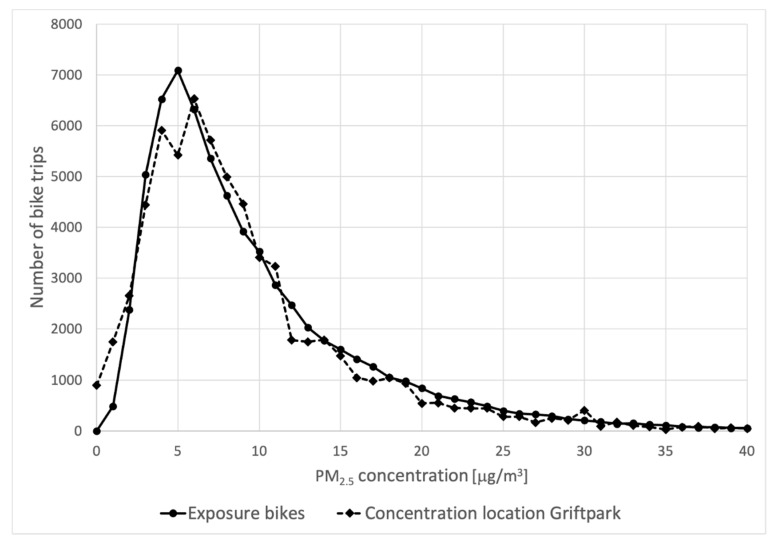
Number of times total PM_2.5_ concentrations were measured by the bicycles and the urban background concentrations in the city of Utrecht (dotted line).

The estimated exposure to (total) PM_2.5_ concentrations during the bike trips is presented in Figure 10. The figure also shows the distribution of PM_2.5_ concentrations at the urban background location in the city of Utrecht. The distributions are quite similar, and both show the familiar lognormal shape.

### 3.2. Estimated Exposure to Traffic-Related PM_2.5_

All measurements were analyzed, taking the 10% percentile of the concentrations measured during the trip as an estimate for the local background concentration. The estimated average PM_2.5_ increase along a bicycle trip is 2.0 μg/m^3^. The resulting average PM_2.5_ concentration contributions due to local traffic are shown in the figure below.

**Figure 11:**
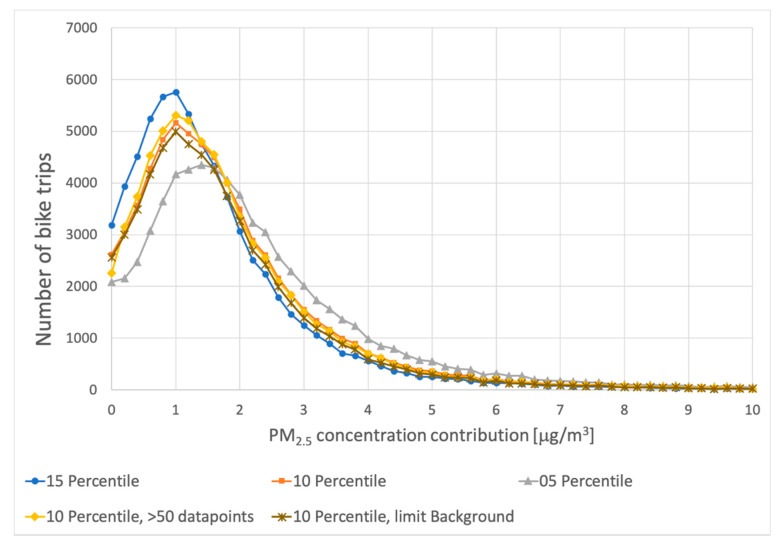
The number of times estimated PM_2.5_ contributions due to road traffic were measured by the bicycles, with different percentiles as background concentration. The “05–10–15“percentiles: estimated contributions due to road traffic using the 0.05–0.10–0.15 percentile of bike measurements as an assumed background; “10 Percentile, 50 points”: estimated contributions due to road traffic using the 0.10 percentile of bike measurements as an assumed background *and* requiring a minimum of 50 datapoints in a bike trip; “10 Percentile, limited”: estimated contributions due to road traffic using the 0.10 percentile of bike measurements as an assumed background *and* excluding hours with relatively fast changing global background in the province.

The number of times different PM_2.5_ contributions of road traffic were measured during bicycle trips is shown in Figure 11. Results are shown for the cases that the 5%, 10% and 15% percentiles of the concentrations measured during bike trips were used as backgrounds. The figure also shows the results in case the 10% percentile is combined with the requirement of having at least 50 valid measurements in a trip, instead of 25. Finally, the figure also shows the estimated PM_2.5_ increase along a bicycle trip when bike trips during hours with relatively large changes in PM_2.5_ concentration in the province are excluded from the analysis (see Section 2.3).

Although the average exposure to emissions of surrounding road traffic is small, in the order of 2.0 μg/m^3^, Figure 11 also shows that about 5% of the trips have a traffic-related exposure of more than 5 μg/m^3^. The choice for the percentile of measurements to be used as an estimate for the background clearly has some influence on the distribution. The overall shape and average exposure do not change significantly. Excluding hours with relatively fast changes in background concentration reduces the average PM_2.5_ increase along a bicycle trip from 2.0 to 1.9 μg/m^3^. The shape of the distribution hardly changes. We conclude that both the effects of requiring more datapoints in a bike trip and excluding hours with changing global PM_2.5_ concentrations have a limited effect on the estimated exposure. The variations in the average PM_2.5_ increase along a bicycle trip depending on the possible choices in the analysis (percentile used, number measurements additional constraints) show a standard deviation of 0.25 μg/m^3^.

**Figure 12:**
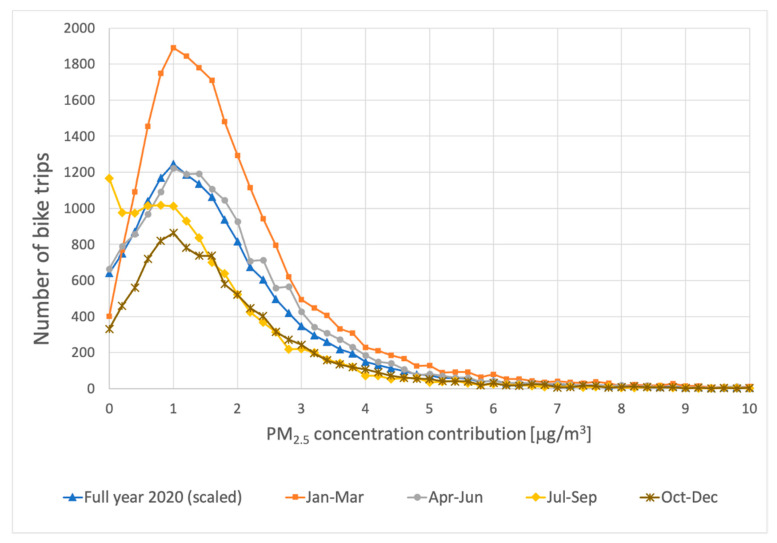
The number of times estimated PM2.5 contributions due to road traffic were measured by the bicycles, for different parts of the year. “Full year 2020 (scaled)”: estimated contributions due to road traffic using the 0.10 percentile of bike measurements as an assumed background and excluding hours with relatively fast changing global background in the province. In order to make the curves easier to compare, the results for the whole of 2020 have been scaled down to 25%. The curves “January–March”, “April–June”, “July–September”, and “October–December” are obtained using data from those months in 2020 only.

The number of bike measurements decreased steadily over the year 2020. However, there are enough measurements to estimate the PM_2.5_ increases along bicycle trips over the course of the year. Figure 12 shows the estimated PM_2.5_ increases along bicycle trips in the periods January–March, April–June, July–September, October–December, 2020. For a better comparison, with the seasonal results, the curve with data from the whole year was scaled down to 25%.

The shapes of the distributions shown in Figure 12 are quite similar. When they are all scaled to the same number of bike trips, only the distribution for the July–September stands out with more bike trips having lower estimated PM_2.5_ contributions due to road traffic. The dispersion of emissions on the road differs in winter and summer, leading to different traffic-related concentrations. Furthermore, with 7.2 μg/m^3^, the average PM_2.5_ concentrations in the province in the summer period (July–September) are almost 2 μg/m^3^ lower than the average in the other months. As a result of the dispersion and absolute concentration levels, the fluctuations of the PM_2.5_ concentrations become very small, probably in the order of the random uncertainty of the sensor measurements. The average estimated PM_2.5_ contributions due to road traffic for the four periods are 2.0 (January–March), 2.0 (April–June), 1.7 (July–September), and 2.1 μg/m^3^ (October–December).

**Figure 13:**
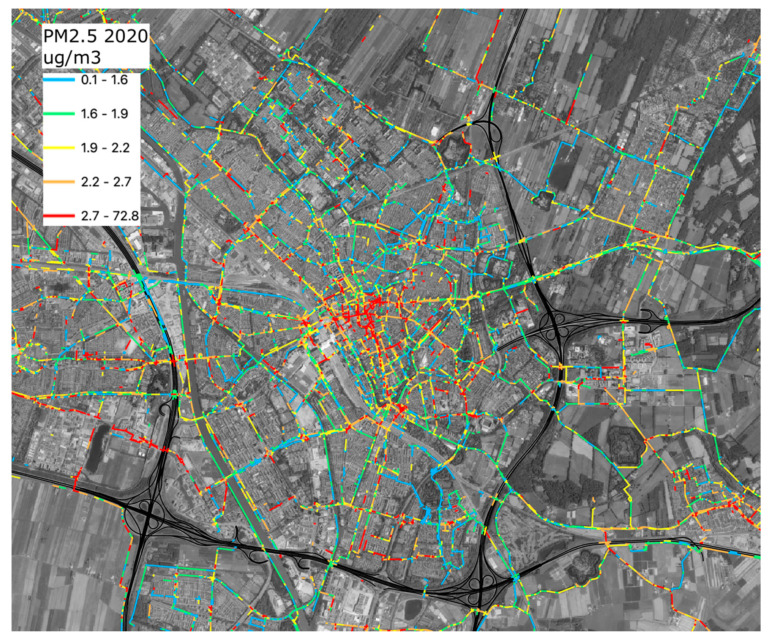
Average PM_2.5_ concentration contributions due to local traffic in the city of Utrecht. All concentrations in μg/m^3^.

**Figure 14:**
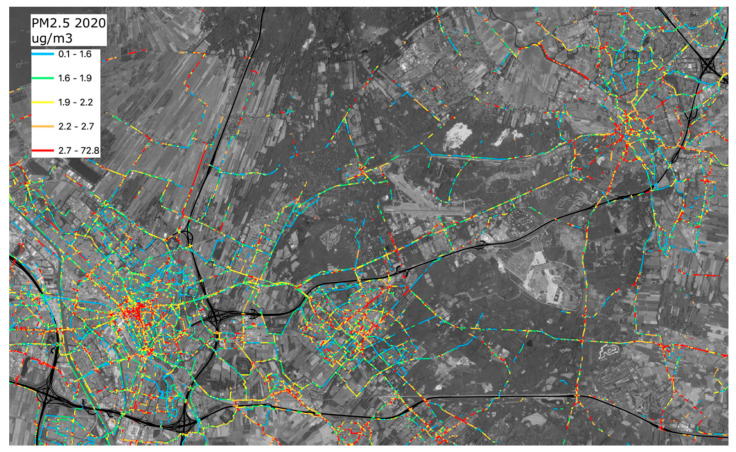
Average PM_2.5_ concentration contributions due to local traffic between the cities of Utrecht (bottom, left) and Amersfoort (top, right). All concentrations in μg/m^3^.

Figure 13 and Figure 14 show the estimated exposure to emissions of surrounding road traffic for parts of the area studied. For every location during a bike trip with a measurement, the estimated traffic-related exposure at that location during that trip is calculated. All these values are stored in a 25 × 25 m^2^ grid covering the whole province of Utrecht. At the end of the analysis, the average traffic-related exposure is calculated for all grid cells and the average exposure is assigned to road segments intersecting those cells. Only road segments in grid cells—in which at least 15 times bikes reported estimated concentration contributions due to traffic—are shown (data must be provided by at least three different bikes). The highways, where cycling is not allowed, are marked in black.

The color scale is classified into five quantiles, each containing 20% of the data values. Blue represents the lowest 20% of concentration contributions, followed by 20% of concentration contributions in green, yellow, orange, and red. There are clear differences between roads in terms of exposure to PM_2.5_ from road traffic. The higher contributions of traffic emissions are usually at locations where many bikes, mopeds, scooters, busses, taxis, and other traffic meet in relatively narrow streets. A large number of locations in the cities where bicycles have to cross busy streets show relatively high traffic contributions. There are also a few routes that show higher concentrations while the local situation (local traffic and geometry) does not suggest these higher values. These seem due to sensors that report slightly higher concentrations than others do, but not high enough to be filtered out.

There are several options to travel between the cities of Utrecht and Amersfoort by bike, shown in the left and the right top of Figure 14. One option is to take a bicycle path that is adjacent to the provincial route between Utrecht and Amersfoort. This path is relatively close to the traffic. Alternatively, bicyclists can take a slightly longer route that roughly follows the train track between the two cities, running to a large extent through woods. There is not much motorized traffic on this route. The results in exposure to traffic emissions between the two routes differ quite substantially. In general, along the routes with low amounts of motorized traffic or where the traffic flows are separated, the exposure to emissions is less than along the provincial route.

We estimated the PM_2.5_ concentration contributions due to local traffic in the city of Utrecht for two different choices for the estimate of the background: the 5% and 15% percentiles. The exposures were classified in 5 quantiles representing relatively low to high exposure, each quantile containing 20% of the data values. From the data, it is clear that the relative exposure along the routes (lower/higher exposure to local emissions) hardly changes when a different percentile is selected for the estimated background. Routes that lead to relatively lower exposure remain the same, independent of the choice made in the analysis. Similarly, the relative hot spots are the same, regardless of the assumptions in the analysis.

## 4. Discussion

This study shows that mobile low-cost sensors mounted on bikes can be a useful way to assess PM_2.5_ exposure along roads and to estimate PM_2.5_ exposure specifically from traffic sources nearby. Our results show that an average cyclist in the province of Utrecht has an estimated traffic-related PM exposure of 2 μg/m^3^, on top of the background concentrations, a small but not insignificant contribution. Moreover, our results show clear differences between busy main roads and quiet back roads. Although the absolute differences in PM_2.5_ concentration between these types of roads are small, the relative difference is consistently present and in the same direction (i.e., measured PM concentrations are higher close to busy roads). Because of the large number of measurements, it is therefore reasonable to suggest that this difference is caused by PM_2.5_ emissions from traffic. Other studies also suggest that it is possible to measure PM from traffic using low-cost sensors [32].

### 4.1. Calibration and Uncertainties

It is well known that low-cost PM sensors have various measurement issues and are not as reliable as official measuring equipment. PM sensors are influenced by, for instance, meteorological conditions, such as relative humidity [33]. Moreover, low-cost sensors might differ in their particle-size selectivity.

Kuula et al. show that the Sensirion SPS30 is able to reasonably measure PM1 and PM_2.5_ fractions but does not measure PM_10_ accurately [31]. Initial tests performed as part of the present project also indicated that PM_10_ values produced by the Sensirion SPS30 are usually very close to the PM_2.5_ values, irrespective of the PM_10_ reported by official measurements. We have therefore decided not to report and use the PM_10_ values at all.

Many authors have tested the calibration of low-cost sensors. Part of the tests on low-cost particulate matter sensors are performed at relatively high concentration, varying the composition of the particles, e.g., [34]. These values are very different from the concentrations encountered in the present study, where the average ambient PM_2.5_ concentrations are in the order of 10 μg/m^3^. Furthermore, the present experiment has no way to assess the size distribution and composition of PM; the calibration has to deal with these types of variations and effects on a real-time basis. Several tests are described in the literature using different schemes to account for the ways environmental conditions influence the behavior of sensors [35]. Instead of using a calibration as a function of separately measured environmental parameters, we decided to test the relatively simple approach described in [15], where the hourly calibration of co-located sensors is assumed to be (sufficiently) representative for the area around those locations. Given the relatively small and uniform nature of the project area, we expected this straightforward method to work sufficiently well. It cannot be excluded that a more elaborate calibration scheme will lead to a better calibration of the data from the Sniffer bikes.

Using co-located sensors to correct for systematic biases on an hourly basis, leads to corrected/calibrated sensor values that are much more in line with official measurements, although the variability in the measured concentrations remains large.

We use the 10% percentile of measured concentrations as an estimate of the background concentration. Using the 5% or 15% percentile does not change our results much, although the estimation of traffic related PM_2.5_ does change. Requiring more measurements in a bike trip also does not influence the results much. We also checked the effect of global variations in PM_2.5_ concentration in the province on the traffic related PM_2.5_, which was also limited. The relative difference in concentrations close to busy and quiet roads is consistently present and in the expected direction. Based on the variations in the traffic related PM_2_._5,_ depending on the specific choices in the analysis, the uncertainty is estimated to be in the order of 0.5 μg/m^3^ for the 95% CI.

There are few exceptions where the estimated contribution of local traffic is high at places where this is not expected based on the type of road. In these cases, there are often very few or just one cyclist cycling this route. We expect our estimations to become better when more cyclists are using a Sniffer bike sensor and are cycling for longer periods of time.

It must be noted that the estimated contributions from the traffic are not representative for yearly average concentration contributions as the bicycle trips are not distributed evenly over the whole day and year. Practically all trips took place between morning and evening (see Figure 8). Moreover, we assume that differences in exposure between busy and quiet roads are due to local traffic. In theory, other local sources close to busy roads might explain the difference, although this is not likely as most other sources of PM_2.5_ will be further away from roads (power plants, wood burning).

### 4.2. Use of Mobile Sensors

The use of mobile air quality sensors makes it possible to estimate exposure to PM_2.5_ while riding on a bike. As a result, it can be used to improve exposure assessments in real-world situations. Several other studies highlight this, showing that pollution levels are generally higher close to breathing levels, compared to levels obtained through stationary measurements [36,37]. Moreover, by mounting air quality sensors on bikes, the willingness to participate in air quality measuring experiments increases significantly. Interest from local governments is also high because the sensor kits also measure additional parameters such as location, speed, temperature and road conditions. In doing so, these sensor kits also contribute to other policy objectives.

Important limitations of using bikes are the limited spread in routes they take and the distribution of the trips over the day. In practice, there are hardly any bike trips in the late evening or night. As a result, it is not possible to compare the averages obtained by the bike to official yearly average values. A further challenge in using mobile sensor measurements is the limited time the sensors are operational. The sensor is only active while moving around, so the total measuring time is low as bike rides are typically around 15 min long. This short operating time makes it more difficult to assess the behavior of an individual sensor. Therefore, the use of mobile sensors would be improved if the sensor is also active while stationary.

Our results suggest that the measured values are often quite high at the start and end of each bike ride. This might be caused by a change in external circumstances, such as the transfer of the sensor from indoor to outdoor, suggesting sensors need a certain amount of initialization time. Therefore, we have not considered the first and last 5% of each bike ride.

### 4.3. Effect of Corona Lock Down

For this study, we used data for the year 2020. Our results might be influenced by the fact that the year 2020 was an atypical year because of coronavirus-related lockdowns. In the Netherlands, several lockdown periods were implemented, most notably at the end of March, mid-October, and at the end of December. During these lockdowns, there was a large reduction in commuting while the number of recreational bike trips increased substantially. As a result, there were significant effects of the lockdown on the emissions and concentration of PM_2.5_ [38]. Therefore, our results might underestimate the contribution of local traffic sources because there was less traffic through the studied period and fewer cyclists may have commuted during rush hours.

### 4.4. Future Directions

There is a lot of interest from local governments to use Sniffer bike sensors, as it has the potential to serve multiple policy objectives. For instance, sensors might be used to characterize not only air pollution exposure, but also heat stress, road quality, and so on. Therefore, we believe the uptake of these sensors will accelerate in the near future. The associated increase in PM_2.5_ measurements will allow us to better estimate exposure to PM_2.5_. Moreover, it would be interesting to include a route planner for the lowest PM_2.5_ exposure and investigate if this leads to meaningful differences in PM exposure over the long-term.

In addition, we believe it is important to have sensors that can be used both stationary and mobile. This allows for a better characterization of sensor behavior and, thus, a better estimation of the quality of individual sensor measurements.

## 5. Conclusions

In this study, we show how mobile air quality sensors (using the Sensirion SPS30) can be used to estimate typical PM_2.5_ concentrations to which cyclists are exposed in the province of Utrecht, the Netherlands.

Our study looked at effects that are well within the uncertainty of individual sensors. Due to the large number of measurements in 2020 (more than 8 million) and the combination of these in many bike trips (almost 68,000), we were able to estimate not only the exposure to absolute PM_2.5_ concentrations during bike trips, but also variations in concentrations during bike trips. We found that the average PM_2.5_ concentration that bicyclists in the project were exposed to in 2020 was 9.9 μg/m^3^. We estimate the average traffic-related exposure in the order of 2 μg/m^3^. During the summer months, we estimate a slightly lower exposure of 1.7 μg/m^3^. In these months, the combination of absolute concentration levels and dispersion of traffic related emissions may lead to fluctuations in concentrations during bike trips that are in the order of the random uncertainty of the sensor and the analysis.

Important limitations of using sensors mounted on bicycles are the limited spread in routes and the uneven distribution of the trips over the day and year. Similar experiments should consider using even more bikes and aim for more diversity in the use of the Sniffer bike sensor units, such as sharing sensor units or enabling use of the sensors while stationary. Quality control should also improve, making sure that sensor kits that produce little or seemingly unrealistic data are quickly checked and, if needed, fixed.

Nevertheless, overall, we conclude that the use of mobile sensors, mounted on bicycles, allows for better characterization of individual exposure to PM_2.5_, while using a bicycle for commuting or for recreational purposes. The mobile PM_2.5_ measurements represents a useful addition to a network of stationary air quality sensors.

## Figures and Tables

**Table 1:** Average sensor values, official measurements, Mean Average Error, Pearson correlation, and Root Mean Square Error for several calibration options. All concentrations are in microgram per cubic meter.

Official Locations	Official Concentration	Raw Sensors Concentration	LSO Sensors Concentration	Raw Sensors Pearson	LSO Sensors Pearson
Kardinaal de Jong	10.3	8.0	8.9	0.86	0.82
Griftpark	9.3	8.3	9.1	0.84	0.81
Breukelen	9.0	7.9	9.1	0.82	0.83
Cabauw	8.8	8.4	10.0	0.73	0.75
Average concentration/value	9.35	8.15	9.29	0.81	0.80
	**Raw Sensors MAE**		**LSO Sensors MAE**	**Raw Sensors RMSE**	**LSO Sensors RMSE**
Kardinaal de Jong	3.8		3.4	4.3	4.5
Griftpark	3.5		3.2	4.7	4.6
Breukelen	3.6		2.9	4.9	4.2
Cabauw	4.1		3.6	6.0	5.4
Average Value	3.8		3.3	5.0	4.7

## Data Availability

All data from the Sniffer bike project are available at www.snuffelfiets.nl/data (accessed on 25 May 2021). All data associated with the figures in this article are available at https://www.samenmetenaanluchtkwaliteit.nl/snuffelfiets/GIS (accessed on 1 June 2021).
